# Trends in prevalence, mortality, health care utilization and health care costs of Swiss IBD patients: a claims data based study of the years 2010, 2012 and 2014

**DOI:** 10.1186/s12876-017-0681-y

**Published:** 2017-12-02

**Authors:** Caroline Bähler, Stephan R. Vavricka, Alain M. Schoepfer, Beat Brüngger, Oliver Reich

**Affiliations:** 1 Department of Health Sciences, Helsana Group, P.O. Box 8081, Zürich, Switzerland; 2 Department Gastroenterology and Hepatology, Stadtspital Triemli, Birmensdorferstrasse 497, 8063 Zürich, Switzerland; 3 Division of Gastroenterology and Hepatology, Centre Hospitalier Universitaire Vaudois/CHUV, Rue du Bugnon 44, 1011 Lausanne, Switzerland

**Keywords:** Inflammatory bowel disease, Prevalence, Mortality, Health care costs

## Abstract

**Background:**

Real-life data on inflammatory bowel disease (IBD) prevalence and costs are scarce. The aims of this study were to provide an overview of the prevalence, mortality, health care utilization and costs of IBD patients in Switzerland in the years 2010, 2012, and 2014.

**Methods:**

Based on claims data of the Helsana-Group, prevalence of IBD was assessed for 2010, 2012 and 2014. Mortality rates, costs (inpatient, outpatient, medication costs) and utilization (visits, hospitalizations) were compared between patients with and without IBD, and between IBD patients treated with and without biologics. Results were extrapolated to the Swiss general population using national census data. Multivariate linear regression was used to identify socio-demographic and regional factors influencing total costs.

**Results:**

The overall extrapolated prevalence rates of IBD were 0.32% in 2010, 0.38% in 2012, and 0.41% in 2014. Mortality rate didn’t differ between the IBD and non-IBD population. Costs increased annually by 6% in IBD versus 2.4% in non-IBD subjects, which was solely due to increased outpatient costs. Almost one-fourth of IBD patients were hospitalized at least once a year. Costs were higher in IBD patients treated with biologics (OR = 3.98, CI: 3.72-4.27, *p* < 0.001) when compared to IBD patients without biologic therapies. Over 70% of the total costs in IBD patients treated with biologics were due to drug costs, compared with 28% in patients without use of biologic therapies, whereas inpatient costs didn’t differ.

**Conclusions:**

The prevalence of IBD seems to be increasing in Switzerland. Outpatient costs increased substantially, while no decrease in inpatient costs was found. Treatment of IBD is more and more based on biologic therapies.

**Electronic supplementary material:**

The online version of this article (10.1186/s12876-017-0681-y) contains supplementary material, which is available to authorized users.

## Background

Inflammatory Bowel Diseases (IBD) are chronic disabling gastrointestinal disorders characterized by a chronic inflammation that can affect the gastrointestinal tract. Ulcerative colitis (UC) and Crohn’s disease (CD) represent the two main forms of IBD, but their etiology and pathophysiology are not yet entirely understood. Genetic susceptibility along with immunological and environmental factors seems to be responsible for the onset of IBD [1–3]. Prevalence estimates range between 0.3% and 0.5%, and are demonstrably higher in Western countries [4–6]. Prevalence and incidence of IBD were shown to have increased over the last years in many, predominantly industrialized, countries [7–10].

IBD may be associated with further physical and mental disorders like iron deficiency or depression compared with the general population [11–14]. These findings result in increased resource utilization in terms of treatment costs, hospitalizations and need for surgeries [15–18]. Moreover, IBD may generate high health care costs as patients are in need of several different medications. The treatment of possible side-effects may further increase the costs. However, a multinational study has found remarkable differences between European countries when assessing health care costs [19]. As claimed by the authors, these differences didn’t result from variations in local pricing, but were rather due to the divergent management of IBD in terms of medical treatment and hospitalization [19].

International guidelines base their recommendations for the treatment of CD [20] and UC [21, 22] on activity and location of the disease. The management plan is also influenced by the disease pattern (relapse frequency, response to previous medications or possible side-effects) and should therefore be individualized [20, 21]. This individualized treatment approach may lead to differences in the practice between clinicians, [21] which may also be true for the management of CD [23, 24].

In Switzerland, only limited data on the prevalence and mortality of IBD is available. Furthermore, no information on the diagnosis (e.g. ICD codes) is available in the ambulatory setting. Data concerning health care costs and utilization in IBD patients who are treated by office-based gastroenterologists and general physicians are scarce. The Swiss IBD Cohort, founded in 2004, mainly consists of patients recruited from (university) hospital divisions and patients treated in the hospital outpatient setting [25, 26]. In addition, many former studies on the economic burden of IBD can be considered outdated due to the introduction of new and effective, but expensive medical therapies.

Switzerland has a mandatory national health insurance system with a cost sharing compulsory basic coverage for each resident consisting of co-payments and deductibles. The height of the deductible is to some extent elective, whereby higher deductibles lead to lower premiums. In contrast, it may increase the amount of out-of-pocket expenses. Managed care models also go along with lower premiums, but they in turn restrain the free choice of physicians. In absence of population-based data on IBD, health care claims data, like the database of the Helsana-Group, are a useful approach to display the prevalence, the health care costs and health care utilization of IBD. Claims data are reliable, practice-based and provide a high level of completeness, independent of the insurance coverage. As the Helsana-Group covers a relatively large and geographically diverse part of the Swiss population, the extrapolated findings can be regarded as representative for Switzerland.

### Aim of the study

The aim of the present study was to provide a comprehensive and updated overview of the prevalence, mortality as well as the health care utilization and costs in IBD patients in Switzerland. In order to assess the time trends, we analyzed the years 2010, 2012, and 2014. Furthermore, we sought to evaluate the proportion of IBD patients treated with biologics, as well as their impact on health care costs and utilization. The comparison of patients treated with and without biologic therapies will provide important additional insights of the real world setting of IBD.

We assume that prevalence and costs of IBD are rising over the observed time period. We hypothesize that outpatient costs are higher in IBD patients with biologic prescriptions compared to those without, while in turn inpatient costs are lower.

## Methods

### Study design

This is a retrospective cohort study of enrollees with and without IBD, who were insured at Helsana-Group in the period between 2010 and 2014. Subjects who died within this period were also included, except in the calculation of the health care costs and the health care utilization. In order to avoid biases due to the cohort effect, we compared three observation periods (using the following three data points: 2010, 2012 and 2014), rather than to follow one cohort for 5 years. Therefore, each of the three study periods had a unique base population, whereby some patients might be included in both or in all three of those periods.

According to the Swiss Federal Law on data protection, this study was exempted from ethics committee approval as all data were anonymized, retrospective, pre-existing, and de-identified in order to protect the privacy of patients, physicians, and hospitals. The study protocol was approved by the Helsana-Group.

### Study population

The Helsana database underlying this study included mandatory health insurance claims from approximately 1.2 million persons per year, covering about 15% of the whole Swiss population. Insured persons were eligible for inclusion if they were obligatory insured by Helsana in the given year (2010, 2012, and 2014, respectively), and if they were at least 1 year old. A total of 1,209,638 individuals in 2010, of 1,206,466 in 2012, and of 1,196,505 persons in 2014 were identified in the Helsana database. In sum 126,972 (3.5%) individuals had to be excluded because they were younger than 12 months of age (1.0%) or due to missing data (2.5%), e.g. individuals living abroad, or nursing home residents whose medical costs were covered by a fixed rate, the reason why no detailed information on the type of medication was available. To calculate health care costs and health care utilization, all decedents (*n* = 34,545) as well as individuals who dropped out of the Helsana-Group (*n* = 46,487) during a given year were excluded in order to have the full range of data for the given year.

### Measures

In absence of epidemiological data including medical diagnoses (e.g. International Classification of Diseases System, ICD), the WHO Anatomical therapeutic chemical (ATC) classification system was used to identify patients with chronic conditions [27]. All prescription drug items are coded and assigned to an ATC code in the Helsana database. Using the pharmacy-based cost group (PCG) model, certain ATC codes can be assigned to different chronic diseases. This mapping approach is frequently used as a reliable method to identify chronically ill patients in administrative data samples if diagnosis information is unavailable [28–30]. We used a modified version of the PCG-Model [31] including 21 chronic conditions in our analysis: acid-related disorders, bone diseases (osteoporosis), cancer, cardiovascular diseases (incl. Hypertension), dementia, diabetes mellitus, epilepsy, glaucoma, gout/hyperuricemia, HIV, hyperlipidemia, iron deficiency anemia, migraine, pain, Parkinson’s disease, psychological disorders (sleep disorders, depression), psychoses, respiratory illness (asthma, COPD), rheumatologic conditions, thyroid disorders, and tuberculosis.

Patients were classified via ATC codes as IBD if they were prescribed 5-aminosalicylic acid (5-ASA: mesalazine and sulfasalazine, prescribed by any physician) and/or if they had at least one anti-inflammatory drug prescription (immunosuppressants, TNF antagonists/biologics, and/or integrin inhibitors) in combination with at least one visit to a gastroenterologist within 1 year. Immunosuppressants included methotrexate, azathioprine, and mercaptopurine, biologics comprised infliximab, adalimumab, golimumab, and certolizumab pegol. The prescription of integrin inhibitors (Vedolizumab) has also been considered for inclusion, although Vedolizumab got its first marketing approval in Switzerland in January 2015. In line with this, we did not find any medical claim for Vedolizumab in any of the observed years. Since 2012, Switzerland has introduced a DRG (“diagnosis related group”) system. The DRG system provides a more detailed analysis of hospitalized IBD patients, which allowed for the calculation of the sensitivity and specificity of the IBD definition used in our study. According to preliminary analysis, the most frequently prescribed medications during the course of 1 year in our hospitalized patients with UC or CD were corticosteroids. However, the inclusion of corticosteroids in the definition would have resulted in a lower specificity.

Further population characteristics were used as covariates. They included sex, age group (1-17, 18-40, 41-60, 61+), language area, type of insurance coverage (managed care model, accident coverage, and deductible class), canton of residence, type of residence, as well as having supplementary insurance and purchasing power of the corresponding zip code as proxies for socioeconomic status.

### Health care costs

We aimed to determine the economic burden of IBD from a social insurance payer perspective including all parts of direct health care costs. Annual total health care costs in IBD patients were obtained from providers’ claims and defined as the total amount of outpatient and inpatient costs per patient per year with mandatory insurance coverage. Inpatient costs contained payments for hospital treatments (over-night stays <24 h), hospitalizations (over-night hospital stays equal to or longer than 24 h), rehabilitation, nursing home, transitional care services and emergency transport services. Costs were not limited to an IBD indication in IBD patients. Costs from the outpatient setting comprised payments for office-based physician visits (primary care physician and specialists), hospital outpatient visits, paramedical visits (e.g. physiotherapists), home care nursing services, medications, laboratory tests and medical devices. Medication costs are presented separately in the Tables. All costs are quoted in Swiss Francs (1 CHF = 1.017 US$; effective August 2016).

The amount of out-of-pocket payments could not be considered in this study because they are not reimbursed by the health insurer. However, these are estimated to be a very small proportion of 1.5% of total payments.

### Health care utilization

Health care utilization is composed of outpatient visits, hospitalizations and of the use of IBD specific medications. Hospitalizations and outpatient visits were both not limited to an IBD indication in IBD patients. In the outpatient setting, visits were defined as direct contacts between patient and physician. The following supplementary measures are provided of outpatient visits in 2014, each per patient/year in those with at least one visit in the given year: the total number of outpatient visits, the number of primary care physician visits (primary care physicians, general internists), the number of specialist visits, and of hospital outpatient visits, as well as the number of different physicians contacted. Hospitalizations were defined as inpatient stays for 24 h or longer. Furthermore, the average length of hospital stay was assessed in those patients with at least one hospitalization.

### Statistical analysis

Using our IBD classification, we calculated the prevalence rates of IBD stratified by age and sex for the years 2010, 2012 and 2014 by dividing the number of patients with IBD by all insured persons of the corresponding strata in the given year. Descriptive statistics were used to show differences between the characteristics of individuals with and without (control group) IBD using fisher’s exact test for dichotomous variables, Wilcoxon rank sum test for continuous, and chi-squared test for categorical variables. Due to the heterogeneity of the Helsana population, it is possible to extrapolate the proportion of patients with IBD for each year to the whole Swiss population by using census data from the Swiss Federal Office of Statistics. The stratification for the extrapolation was carried out by age group, sex and 26 cantons of residence using the R package ‘survey’ [32]. We further calculated the annual all-cause mortality in patients with IBD for the three study periods. Direct health care costs and health care utilization are presented separately for patients with and without IBD. Mortality rates, total health care costs and health care utilization were also extrapolated to the Swiss general population.

Multivariate logistic (prevalence and mortality) or linear (health care costs and utilization) regression modeling, including year, sex, age group and canton of residence as independent factors, was conducted for the description of time trends. Additional, linear multivariate regression modeling was performed to predict the total costs in IBD patients, adjusting for the following influencing factors: age group, sex, treated comorbid conditions, regional variables, health insurance plan, as well as socioeconomic status. In the presence of a skewed distribution of costs, log transformation was used. Finally, differences within IBD patients with and without the use of biologics were analyzed regarding health care utilization and costs, thereby again adjusting for the above influencing factors. Analyses were conducted using R statistics, version 3.2.0 [33].

## Results

The number of persons included in the final study sample comprised 1,170,913 in 2010, 1,163,351 in 2012, and 1,151,373 in 2014. Overall, 51.7% of the individuals were women. The median (IQR) age was 43.0 (35) in men and 46.0 (38) in women. The age distribution of men and women in the three study populations is shown in Additional file 1: Table S1.

### Prevalence

Overall, 0.37% of the extrapolated Swiss population were defined as having IBD between 2010 and 2014. The percentages of Swiss IBD patients totaled up to 0.32% in 2010, 0.38% in 2012, and 0.41% in 2014. Prevalence estimates of IBD extrapolated to the general Swiss population by age, sex, and canton of residence with the corresponding confidence intervals (CI) for the three given years are shown in Table 1.

**Table 1:** Prevalence estimates of IBD extrapolated to the general Swiss population for the years 2010, 2012, and 2014

	2010	2012	2014
Prevalence /100,000 (CI)			
Total	318 (308-329)	376 (365-388)	408 (396-420)
Men (all ages)	284 (270-298)	338 (322-353)	372 (356-388)
1-17	24 (13-34)	25 (14-37)	19 (10-29)
18-40	213 (191-236)	255 (230-281)	303 (276-330)
41-60	373 (343-403)	447 (412-481)	469 (435-504)
60+	495 (458-532)	567 (527-606)	627 (585-669)
Women (all ages)	352 (337-367)	414 (397-430)	443 (426-460)
1-17	20 (11-30)	27 (16-38)	26 (16-37)
18-40	299 (273-326)	321 (293-349)	381 (350-412)
41-60	445 (413-478)	552 (514-590)	564 (526-602)
60+	535 (502-568)	616 (580-653)	648 (611-685)

The differences in the characteristics between the IBD and the non-IBD sample are shown in Table 2. Prevalence rates were significantly higher in the German (0.40%) and the Italian speaking part (0.42%) of Switzerland, compared with the French speaking part (0.31%). We found higher prevalence estimates of IBD in urban compared with rural areas. Concerning the health insurance plan, IBD patients were less frequently enrolled in a managed care model and more frequently had supplementary private hospital insurance coverage.

**Table 2:** Characteristics of the study population in 2014

n (%)	Total	IBD	Non-IBD	*p* ^a^
	*n* = 1,125,050	*n* = 4812	*n* = 1,120,238	
Female sex	581,598 (51.7)	2695 (56.0)	578,903 (51.7)	<0.001
Age				
Total (mean, SD)	44.0 (23.5)	54.8 (18.0)	43.9 (23.5)	<0.001
Men (mean, SD)	42.3 (22.8)	54.2 (17.5)	42.2 (22.8)	
Women (mean, SD)	45.5 (24.0)	55.4 (18.5)	45.5 (24.0)	
Language area^b^				
German	840,900 (74.7)	3778 (78.5)	837,122 (74.7)	<0.001
French	209,719 (18.6)	687 (14.3)	209,032 (18.7)	
Italian	74,413 (6.6)	347 (7.2)	74,066 (6.6)	
Type of residence (urban area)	874,640 (77.7)	3799 (78.9)	870,841 (77.7)	0.04
Chronic conditions (median, IQR)	0.0 (2.0)	3.0 (3.0)	0.0 (2.0)	<0.001
Home care nursing dependency	30,669 (2.7)	285 (5.9)	30,384 (2.7)	<0.001
Health insurance plan				
Managed care	649,992 (57.8)	2340 (48.6)	647,652 (57.8)	<0.001
Higher deductible	308,642 (27.4)	522 (10.8)	308,120 (27.5)	<0.001
Accident coverage	677,107 (60.2)	2871 (60.0)	674,236 (60.2)	ns
Supplementary hospital insurance	208,661 (18.5)	1020 (21.2)	207,641 (18.5)	<0.001
Purchasing power				
1 (high)	224,750 (20.0)	956 (19.9)	223,794 (20.0)	ns
2	223,464 (19.9)	942 (19.6)	222,522 (19.9)	
3	224,857 (20.0)	961 (20.0)	223,896 (20.0)	
4	222,979 (19.8)	984 (20.4)	221,995 (19.8)	
5 (low)	229,000 (20.4)	969 (20.1)	228,031 (20.4)	

^a^ *p-values,* assigning the differences between the IBD and non-IBD populations, were calculated using fisher’s exact test for dichotomous variables, using Wilcoxon rank sum test for continuous, and using chi-squared test for categorical variables; ns = not significant

^b^Rhaeto-Romanic area is assigned to the German area

Using logistic regression modeling, we found that women were more likely to have an IBD diagnosis: the overall adjusted sex-difference in the proportion of IBD patients was OR 1.16 (CI: 1.12-1.20, *p* < 0.001), when taking age group and canton of residence into account. The prevalence estimates increased strongly with older age, in men (OR = 25.82, CI: 20.29-33.51, *p* < 0.001), and women (OR = 23.11, CI: 18.37-29.62, *p* < 0.001), compared with their youngest counterparts. When comparing three time points within 5 years, we found an overall annual increase in the prevalence of 6% (OR = 1.06, CI: 1.05-1.07, *p* < 0.001) between 2010 and 2014, allowing for the varying distribution of age, sex and the canton of residence of the individuals.

Based on the study population, the most frequently prescribed medications in patients with IBD were 5-ASA. However, the proportion of patients with at least one claim for mesalazine or sulfasalazine decreased, whereas the prescription of immunosuppressants and biologics increased in the given time span (Table 3).

**Table 3:** Percentage of IBD patients with at least one of the listed medical claims (*n* = 13,451)

%	Total	2010	2012	2014
5-ASA^a^	87.4	96.8	84.8	82.2
Immunosuppressants^b^	23.2	18.7	24.4	25.9
Biologics^c^	9.7	5.3	10.2	12.8
Steroids^d^	35.0	32.8	36.5	35.3

^a^mesalazine, sulfasalazine

^b^methotrexate, azathioprine, mercaptopurine

^c^infliximab, adalimumab, golimumab, certolizumab pegol

^d^prednisone, prednisolone, methylprednisolone, budesonide

In a sub-analysis we compared the prevalence rate estimated by means of the IBD definition described above (using ATC codes) with a main listing of ICD-10 diagnosis of UC or CD (K50, K51) in hospitalized patients in 2014. We found a sensitivity of 60.5% and a specificity of 99.1%.

### All-cause mortality

Overall, 1.3% of IBD patients died between 2010 and 2014. The proportion of decedents in the non-IBD cohort amounted to 0.8%. However, this difference is not statistically significant when adjusting for age and sex (OR = 1.09; CI: 0.95-1.24, *p* = 0.234). The median (IQR) age was 81.0 (13.8) years in decedents with and 83.0 (16.0) years in decedents without IBD (*p* = 0.001).

The mortality estimates of male IBD patients ranged from 1.2% (0.8-1.6%) in 2010 and 1.2% (0.7-1.5%) in 2012 to 1.8% (1.3-2.3%) in 2014, when extrapolated to the Swiss general population. The corresponding estimates in women were 1.1% (0.8-1.5%), 1.4% (1.0-1.8%), and 1.0% (0.7-1.4%), respectively. There was no significant change in all-cause mortality rate over the observed time span, when controlling for age group, sex and the canton of residence: OR = 1.02 (CI: 0.94-1.11, *p* = 0.662). However, as the number of decedent IBD patients was rather small, these data have to be interpreted with caution.

### Health care costs

Median (CI) total health care costs per IBD patient extrapolated to the Swiss population were CHF 5390 (5170-5610), CHF 6090 (5800-6370), and CHF 6810 (6520-7100) in 2010, 2012, and 2014, respectively, compared with CHF 750 (740-750), CHF 780 (780-790), and CHF 870 (860-870), in non-IBD patients (Table 4). Nearly one fifth of the non-IBD population had no costs at all in the given year. Overall, total direct costs incurred by IBD patients were substantially higher compared to the non-IBD sample (OR = 4.97, CI: 4.85-5.10, *p* < 0.001), having adjusted for age, sex, canton of residence, and year of observation. Additional file 2: Table S2 presents the median total health care costs of the IBD and the non-IBD sample for the years 2010-2014, extrapolated to the Swiss population, by sex and age group. In total, female IBD patients incurred higher costs compared to male patients throughout the three observed time points (OR = 1.62, CI: 1.61-1.62, *p* < 0.001), having adjusted for age, canton of residence, and year of observation. The overall costs in patients aged 17 years and younger shown in Additional file 2: Table S2 should be interpreted with caution due to low sample sizes.

**Table 4:** The extrapolated health care costs (in Swiss Francs) in IBD versus non-IBD patients between 2010 and 2014

Mean (CI, median)	IBD	non-IBD	*p* ^a^
2010			
Total	9590 (9210-9970, 5390)	3160 (3140-3170, 750)	<0.001
Inpatient	2040 (1830-2240, 0)	890 (880-890, 0)	<0.001
Outpatient	3920 (3790-4040, 2810)	1510 (1500-1510, 490)	<0.001
Medication	3630 (3430-3840, 1700)	760 (760-770, 100)	<0.001
2012			
Total	11,680 (11,230-12,140, 6090)	3380 (3360-3390, 780)	<0.001
Inpatient	2080 (1860-2300, 0)	950 (940-960, 0)	<0.001
Outpatient	4470 (4300-4640, 3110)	1630 (1630-1640, 520)	<0.001
Medication	5130 (4840-5430, 1900)	790 (790-800, 110)	<0.001
2014			
Total	12,790 (12,270-13,300, 6810)	3540 (3520-3560, 870)	<0.001
Inpatient	2150 (1870-2430, 0)	910 (900-920, 0)	<0.001
Outpatient	5280 (5070-5490, 3510)	1830 (1830-1840, 610)	<0.001
Medication	5360 (5100-5620, 1900)	800 (790-800, 110)	<0.001

^a^ *p-values* were calculated using multivariate linear regression (adjusted for age group, sex, and canton of residence)

The extrapolated mean total health care costs incurred by IBD patients were about four times higher in 2014 compared with those of the non-IBD Swiss population (Table 4). Extrapolated medication costs were even more than 6 times higher in IBD patients in this same year. In IBD patients, the proportion of medication costs as well as of the other outpatient services amounted to 42% each, whereas only 16% of the costs were due to hospital admissions in 2014. Four years earlier, the proportion of inpatient costs made up 21% of the total costs. A more detailed description of the health care costs in the IBD versus the non-IBD sample are provided in Additional file 3: Table S3.

**Fig. 1:**
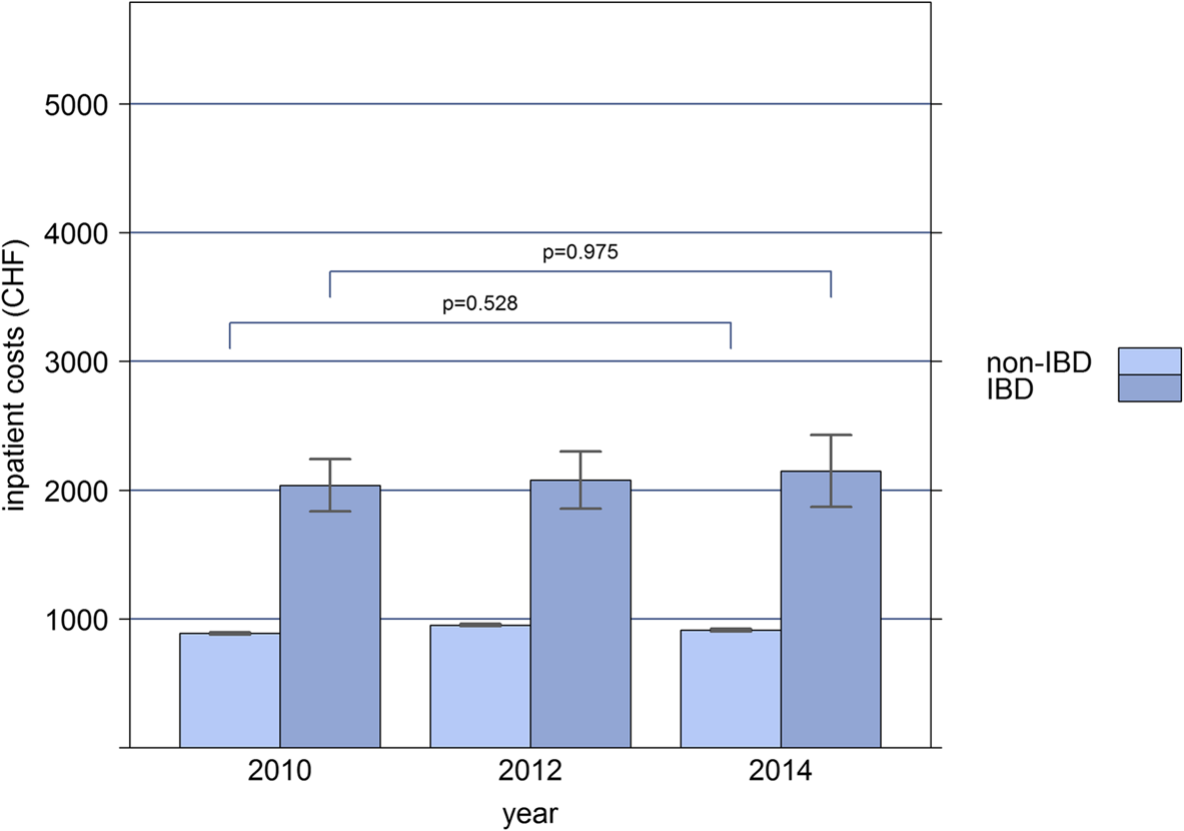
Bar-plot of the mean annual inpatient costs (in Swiss Francs) of IBD and non-IBD patients, extrapolated to the general Swiss population for the years 2010, 2012, and 2014, respectively

**Fig. 2:**
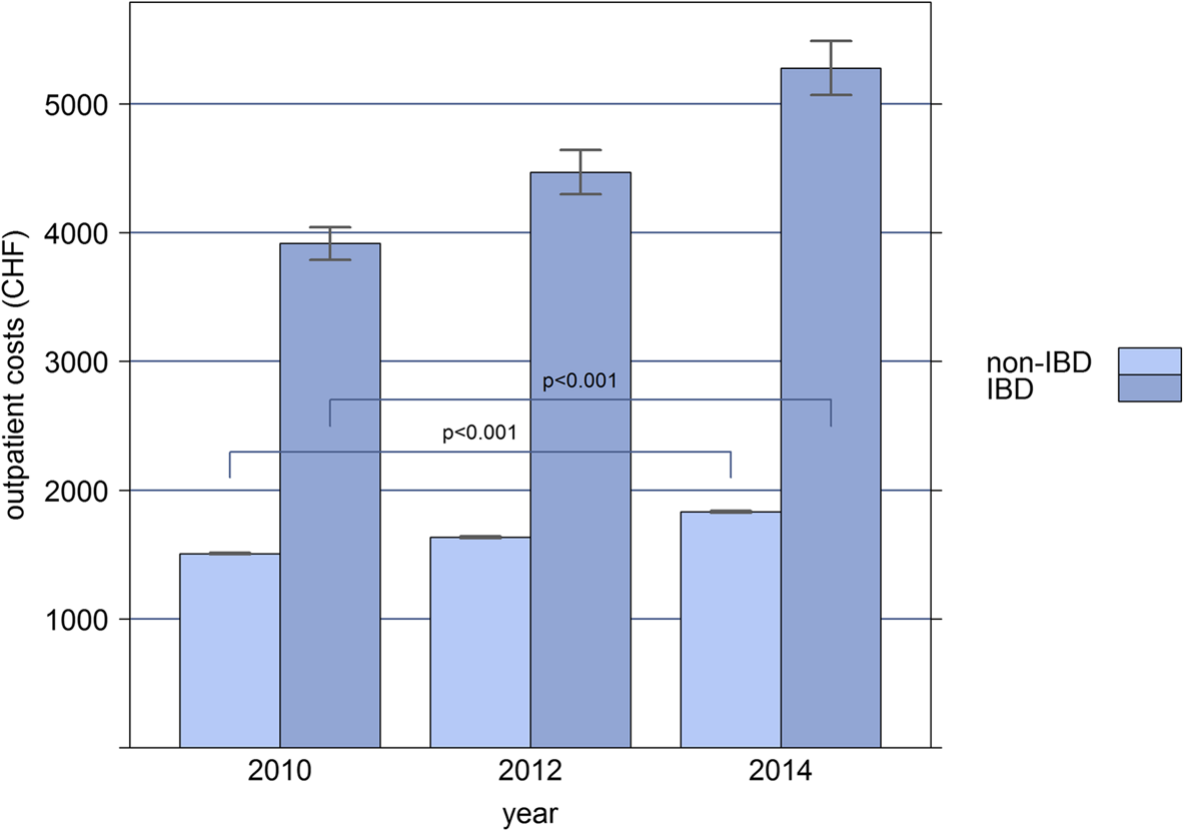
Bar-plot of the mean annual outpatient costs (in Swiss Francs) of IBD and non-IBD patients, extrapolated to the general Swiss population for the years 2010, 2012, and 2014, respectively

**Fig. 3:**
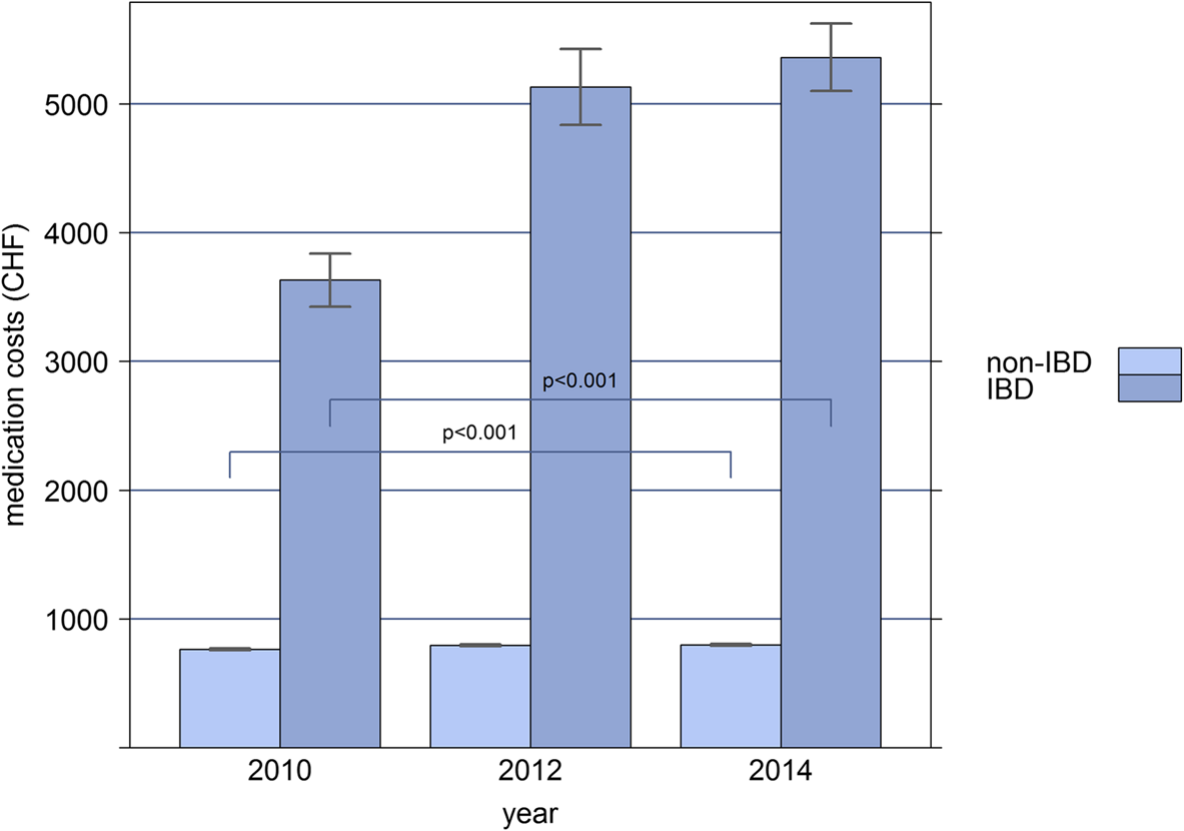
Bar-plot of the mean annual medication costs (in Swiss Francs) of IBD and non-IBD patients, extrapolated to the general Swiss population for the years 2010, 2012, and 2014, respectively

Based on our analysis of three time points within 5 years, total health care costs showed an annual increase of 2.4% (OR = 1.024, CI: 1.023-1.025, *p* < 0.001) in the non-IBD sample, and of 6.0% (*p* < 0.001) in IBD patients, when age, sex and canton of residence were taken into account. In IBD patients, the increase was solely due to increased costs of outpatient services (OR = 1.06, CI: 1.05-1.08, *p* < 0.001), and medication costs (OR = 1.05, CI: 1.04-1.06, *p* < 0.001), while there was no significant change in inpatient costs between 2010 and 2014 (OR = 1.00, CI: 0.98-1.02, *p* = 0.975). Similarly, in non-IBD patients, outpatient costs (OR = 1.036, CI: 1.035-1.037, *p* < 0.001), and to a lesser extent, medication costs (OR = 1.002, CI: 1.001-1.002, *p* < 0.001) increased, while inpatient costs remained stable (OR = 1.00, CI: 1.00-1.01, *p* = 0.528; Figs. 1, 2 and 3).

Further analyses were conducted to examine the association between the preselected socio-demographic, clinical and regional predictors and the total health care costs in IBD patients (Table 5). Total health care costs were lower in patients aged 60 years and older compared to patients aged 1-17 years, in both men and women. Less surprisingly, the number of chronic conditions and nursing dependency led to increased total costs. IBD patients in managed care model had 13% lower total costs compared with those in traditional model. In the Italian speaking part (as compared with the German speaking part), costs of IBD patients were associated with a decrease of 14%.

**Table 5:** Multiple linear regression model on the total health care costs in IBD patients in 2014 (*n* = 4812)

	β (95% CI)	*p*
Age group by males		
1-17 (male)	1.00	
18-40 (male)	0.59 (0.39-0.91)	0.016
41-60 (male)	0.47 (0.30-0.71)	<0.001
60+ (male)	0.44 (0.29-0.67)	<0.001
Age group by females		
1-17 (female)	1.00	
18-40 (female)	1.04 (0.72-1.49)	0.840
41-60 (female)	0.79 (0.55-1.13)	0.195
60+ (female)	0.59 (0.41-0.85)	0.004
Number of chronic conditions	1.31 (1.29-1.33)	<0.001
Nursing dependency	1.89 (1.69-2.12)	<0.001
Language area		
German	1.00	
French	1.03 (0.96-1.11)	0.375
Italian	0.86 (0.78-0.95)	0.003
Health insurance plan		
Higher deductible class	0.65 (0.60-0.71)	<0.001
Accident coverage	1.10 (1.03-1.18)	0.004
Managed care	0.87 (0.83-0.92)	<0.001
Supplementary insurance	1.06 (0.99-1.14)	0.061
R^2^	0.337	

The type of residence was not included in the final model as urbanity was no longer significant once language area was taken into account. No association with total costs was found for purchasing power and supplementary private hospital insurance.

### Health care utilization

Overall, the number of individuals demanding health care was significantly higher in the IBD compared to the non-IBD-sample. In 2010, 97.6% of the IBD patients and 74.0% of the non-IBD sample had at least one physician visit. The corresponding proportions were 98.0% and 76.4% in 2012, and 98.3% and 77.7% in 2014, respectively. The median (CI) number of visits per IBD patient in the extrapolated Swiss population were 13 (12-14), 15 (14-16), and 15 (15-16) in 2010, 2012, and 2014, respectively, compared with a median number of 3 (3-3), 4 (4-4), and 4 (4-4) in the non-IBD sample in the corresponding years. IBD patients had a substantially higher number of annual physician visits (OR = 2.75, CI: 2.70-2.80, *p* < 0.001), compared to those without IBD, after adjustments for age, sex, canton of residence, and year of observation. Female IBD patients have contacted physicians more frequently (adjusted OR = 1.30, CI: 1.26-1.33, *p* < 0.001) than male IBD patients.

The total number of visits increased by 3.7% (OR = 1.04, CI: 1.03-1.05, *p* < 0.001) in IBD-patients over the observed time period, when age, sex and canton of residence were taken into account. Differences between IBD and non-IBD patients regarding the number of visits to primary care physicians, to specialists and the number of hospital outpatient visits are provided in Additional file 4: Table S4. The greatest difference regarding the number of visits referred to specialist visits.

The proportion of IBD patients with at least one hospitalization in a given year ranged between 23.1% and 24.2% during the observed time span. In contrast, only 10.8% to 10.9% of patients without IBD were hospitalized at least once in the corresponding years. The median (IQR) and mean (SD) length of hospital stay amounted to 7 (14) and 15.2 (23.4), respectively 5 (9) and 14.0 (29.5) days in those hospitalized patients.

### Sub-analyses of IBD patients in 2014

Finally, we analyzed health care costs and utilization in IBD patients, thereby comparing individuals with and without any prescription for biologics (infliximab, adalimumab, golimumab, certolizumab pegol). Of the 4812 IBD patients in 2014, 620 (12.9%) were treated with biologics. Characteristics of the two groups are shown in Table 6. Biologics were more frequently prescribed in younger patients, and in patients living in the French speaking part of Switzerland, as well as in those living in urban areas.

**Table 6:** Characteristics of IBD patients treated with or without biologics in 2014 (*n* = 4812)

n (%)	IBD (total)	No Biologics	Biologics	*p* ^a^
	*n* = 4812	*n* = 4192	*n* = 620	
Female sex	2695 (56.0)	2461 (56.1)	234 (55.1)	ns
Age				
Total (mean, SD)	54.8 (18.0)	55.9 (17.9)	43.6 (15.7)	<0.001
Men (mean, SD)	54.2 (17.5)	55.4 (17.2)	41.8 (15.4)	
Women (mean, SD)	55.4 (18.5)	56.4 (18.4)	45.1 (15.8)	
Language area^b^				
German	3778 (78.5)	3473 (79.2)	305 (71.8)	<0.001
French	687 (14.3)	582 (13.3)	105 (24.7)	
Italian	347 (7.2)	332 (7.6)	15 (3.5)	
Type of residence (urban area)	3799 (78.9)	3446 (78.6)	353 (83.1)	0.03
Health insurance plan				
Managed care	2340 (48.6)	2154 (49.1)	186 (43.8)	0.04
Higher deductible	522 (10.8)	509 (11.6)	13 (3.1)	<0.001
Accident coverage	2871 (59.7)	2660 (60.6)	211 (49.7)	<0.001
Supplementary hospital insurance	1020 (21.2)	948 (21.6)	72 (16.9)	0.03
Home care nursing dependency	285 (5.9)	263 (6.0)	22 (5.2)	<0.001

^a^ *p-values,* assigning the differences between IBD patients treated with and without biologics, were calculated using fisher’s exact test for dichotomous variables, using Wilcoxon rank sum test for continuous, and using chi-squared test for categorical variables; ns = not significant

The total costs incurred by patients treated with biologics were almost three times higher compared with patients without biologics (Table 7). In the multivariate regression model controlling for socio-demographic (age, sex, and type of insurance coverage), clinical (number of chronic conditions, nursing dependency) and regional (language area, type of residence) factors, the costs were even four times higher (OR = 3.98, CI: 3.72-4.27, *p* < 0.001). More than 70% of the total costs were due to medication costs in patients with biologics, compared with 28% in patients without biologics. Interestingly, all cost groups differ significantly between the two groups except for the inpatient costs.

**Table 7:** Health care costs (in Swiss Francs) of IBD patients treated with or without biologics in 2014 (*n* = 4812)

Mean (SD, median)	No Biologics	Biologics	*p* ^a^
Total	10,437 (15,146, 5833)	28,265 (12,970, 25,779)	<0.001
Inpatient	2308 (8340, 0)	1887 (4790, 0)	0.3
Outpatient	5163 (6912, 3368)	6303 (4830, 4956)	<0.001
Primary care physicians	635 (777, 417)	551 (769, 285)	<0.001
Specialists	1516 (1854, 941)	2026 (2191, 1509)	<0.001
Others (e.g. paramedical)	3012 (6044, 1401)	3726 (3630, 2692)	<0.001
Medications	2966 (5464, 1644)	20,075 (9486, 19,316)	<0.001

^a^ *p-values* were calculated using Wilcoxon rank sum test

Only 1.7% of the IBD patients without biologics and none of the patients with biologics did not have any consultation in 2014. The median (IQR) total number of visits amounted to 22 (19) in IBD-patients with and to 15 (18) in patients without biologics in those with at least one consultation (*p* < 0.001). The median (IQR) number of visits to primary care physicians and to specialists was 4 (8) and 9 (12) in IBD-patients with, and 5 (9) and 5 (8) in IBD patients without biologics, respectively.

The proportion of IBD patients with at least one hospital admission in 2014 was 25.6% and 22.1% in patients with and without biologics, respectively (*p* = 0.06). The median (IQR) and mean (SD) length of hospital stay amounted to 7 (11) and 12 (13) in those treated with biologics, and 8 (15) and 17 (27) days to in those treated without biologics. Thus, no significant differences regarding the inpatient setting were found between the two groups of IBD patients.

## Discussion

### Prevalence and mortality rates of IBD

Based on our analysis of three time points within 5 years, we found an overall extrapolated prevalence rate of IBD of 0.37%, whereby the rate increased from 0.32% in 2010, to 0.38% in 2012, and to 0.41% in 2014. This is comparable to estimates in Sweden (0.35%), Finland (0.44%), the US (0.44%) and Canada (0.5%) [4–6, 34]. According to a recently published review by Burisch and colleagues, [15] 0.3% of the population had IBD in Europe. Our extrapolated prevalence rate was slightly lower than that of a large German insurance-based cohort with 493/100,000 actively treated IBD patients in 2010 (age- and sex-standardized to the German population) [35]. According to a previous Swiss study, [26] the estimated prevalence rate for Switzerland was 206/100,000. The incidence of both, CD and UC, were shown to have increased in Switzerland since then, [25] which was also observed in other countries [9]. In a large US cohort study, the prevalence rates in 2009 were 241/100,000 for CD and 263/100,000 for UC adult patients (20 years and older), respectively. These rates correspond to an increase compared with the years 2003/2004, where 201/100,000 were estimated to have CD and 238/100,000 were estimated to have UC [5, 7]. In their claims data based study, patients with at least one IBD-specific medication, combined with at least one claim for CD or UC, were also included in their case definition [7].

According to our analysis, IBD was more prevalent in women compared to men (403/100,000 vs. 331/100,000), and was lower in persons aged 17 years and younger (24/100,000) as compared with all other age groups. Similarly, a higher rate of female compared to male IBD patients was reported for the Swiss IBD Cohort, [36, 37] as well as for other countries in Europe [17, 35]. In US children, prevalence rates were estimated to be 43/100,000 for CD and 28/100,000 for UC, respectively, which is slightly higher than in our findings [38]. However, our results need to be interpreted with caution, as the sensitivity of our defined IBD identification algorithm is relatively small, whereas the estimated specificity was found to be high. In other words, we possibly underestimate the number of patients with IBD. Reasons for the comparably low sensitivity might, firstly, be due to the fact that we were unable to detect patients with mild disease who weren’t treated with one of the defined medications [37]. Secondly, there might be incident cases without a history of IBD-related medications. Thirdly, when medications are administered during hospitalization, coding for this treatment is missing by means of our data. Lastly, as the disease is characterized by relapsing intestinal inflammation, the medical treatment varies. However, in absence of clinical data like ICD codes, the use of highly reliable medical claims to estimate the burden of a chronic disease is common. A previous validation study on IBD based on an administrative database has found a sensitivity of 88.9% and 89.2% and a specificity of 91.2% and 89.8% for CD when comparing self-reports with chart-reviews, and a sensitivity of 87.7% and 74.4% and a specificity of 91.3% and 93.7% for UC, respectively [39].

Looking solely at medical claims, the percentage of IBD patients with a drug prescription amounted to 19% for immunosuppressants and 5% for biologics in our study in 2010. The corresponding figures were 19% and 3% in a Swedish study [34]. In contrast, their prescription rates for aminosalicylates were considerably lower [34]. In a Dutch study, 30.1% of the IBD patients received immunosuppressants, 15.1% received biologics, and 39.7% were on 5-ASA in 2011 (as compared with 84.8% of patients with 5-ASA in 2012 in our study) [17].

We found no increased mortality ratio in patients with IBD compared to the non-IBD cohort. This is in line with previous studies in the Netherlands, Finland or North America [40–42]. Further previous studies assessing mortality rates in IBD patients found a higher mortality ratio in CD, but not UC patients, for example in the US, Denmark, or in overall Europe [15, 43, 44]. In contrast, slightly higher rates were found for overall IBD patients, [45–47] that were commonly more pronounced in younger patients [43, 46]. Mortality rates were shown to decrease over the last decades, especially in hospitalized patients [48].

### Health care costs and health care utilization

Based on our findings, the extrapolated median (mean) total health care costs per IBD patient in Switzerland were CHF 5390 (9590) in 2010, CHF 6090 (11,680) in 2012, and costs increased to CHF 6810 (12,790) in 2014. According to a review by Yu et al. [49] published in 2008, estimated annual direct medical costs per CD patient were approximately 18,000 to 19,000 US$ in the US, and approximately 4000 to 10,000 US$ (converted) in other Western countries. Similarly, lower costs for the treatment of CD and UC in Europe compared to the US have been shown in the review by Odes [50]. From 1999 to 2005, annual medical costs per patient were estimated to be 18,963 in CD patients versus 5300 US$ in controls, and 15,020 in UC patients versus 4982 US$ in controls in the US [51]. The calculated increase in total costs in our study is in line with international findings. Based on health claims data, the average annual medical costs per patient with CD and UC amounted to 6561 US$ and 1488 US$, respectively, in the US in 1990 [52]. Thirteen to fourteen years later, the direct medical costs of CD and UC per patient per year were estimated to be 10,952 US$ (with CD associated treatment costs of US$ 8265) and 7948 US$ (with UC associated treatment costs of US$ 5066) from a social insurance payer perspective, respectively [18].

Based on our study findings, women incurred higher total health care costs than men. And in the multivariate regression model on total costs in IBD patients, costs were highest in the youngest age group. However, the confidence interval in the youngest patients are wide, suggesting great variance and heterogeneity in the management of the youngest patients.

In contrast to our findings, no significant sex-related differences were found concerning total health care costs in a large US study [18]. In that same study, younger patients (aged <20 years) were shown to incur higher mean annual health care costs compared to their older counterparts, especially in CD patients [18, 53]. The higher costs in the youngest age group were explained 1) by a higher number of incident cases in this age group, 2) by a more severe course of disease when IBD is diagnosed at young age, and moreover 3) by a possibly more progressive treatment pattern of pediatric gastroenterologists [18].

There has been a shift from inpatient to outpatient costs in recent years, due to the introduction of new medical therapies like biologics, mainly in patients with CD [16, 18, 50]. This is comparable to our results, where outpatient costs made up 79% in 2010 and 84% in 2014. However, the increase in outpatient costs might also be a consequence of the introduction of the Swiss DRG in 2012, which might have led to a shift of treatments and dispensing of medications from the inpatient to the outpatient setting. In earlier studies conducted in the US, [49, 52] and in European countries, [19, 49] more than half of the total costs were attributable to inpatient costs. Medical and surgical hospitalization made up a little more than 30% in a US study conducted between 2003 and 2004 [18]. In a recent Dutch study [17] that looked at the societal perspective of the costs of IBD, medication costs accounted for 71% of the total costs in CD and for 59% in UC patients. Inpatient costs made up as little as approximately 20% of the total costs in 2011 [17].

In 2014, 98.3% of IBD patients in our sample had at least one physician visit. The extrapolated median (IQR) number of visits per IBD patient was 15 (15-16) in 2014, compared to 4 (4-4) in the non-IBD sample, whereby the greatest differences referred to specialist visits. The proportion of IBD patients with at least one hospital admission in 2014 was nearly 24%. The median (mean) length of hospital stay amounted to 7 (16) days in those patients.

In a previous Swiss study, [37] the mean (± SD) number of outpatient visits was 2.1 (± 3.6) and 2.3 (± 3.2) in UC and CD patients during the 3-months follow-up, respectively, whereby patients more frequently consulted a specialist rather than a primary care physician in their analyses as well. However, they only investigated IBD-related resource consumption, whereas we didn’t consider the reason for hospitalization or consultation. Almost 14% of the IBD patients were hospitalized for at least 1 day in their 12-months follow-up [37]. Their calculated mean (± SD) number of days in hospital was 1.5 (± 6.1) and 2.0 (± 8.8) in UC and CD patients, respectively. Yet, they didn’t exclude patients without any consultation or hospitalization in their study. Similarly, about 14% of the IBD patients were hospitalized in the UK [54]. In a Swedish study, 24% of the IBD patients had undergone at least one major IBD-related surgery during the study period [34]. According to a recent review, the hospitalization rates varied considerably between European countries [15]. The proportion of IBD patients with surgery ranged between 0.5% in an Hungarian and 35% in a Danish cohort during 1 year of observation. To conclude, the diversity in the definition or classification of visits does not allow always for direct comparison of the results found in our study with previous study findings.

### Comparison of IBD patients with and without the use of biologics

In 2014, 13% of all IBD patients in the Helsana cohort received biologics. They incurred four times higher total costs compared with patients without such a prescription. About 70% of the costs were attributable to medication costs. But the higher medication costs were not compensated by lower inpatient costs. However, these results need to be interpreted with caution as we could not take the year of diagnosis or the disease severity into account. It is known that patients treated with biologics are mostly affected by a more severe disease course. Furthermore, inpatient costs were not limited to an IBD-related hospitalization.

Thus, similar results were found by Kappelman et al. [18] in 2003 and 2004. According to their study, biologics (namely infliximab) were the most expensive medications in CD patients, whereas aminosalicylates were the most costly drugs in UC patients. A higher consumption of health care resources in patients treated with biologics has also been found in further studies [37, 55]. The higher resource consumption mainly occurred in the first 1-3 years after initiation of the treatment in those analyses, which might be another reason for the higher costs in young patients reported in our study.

In contrast, a Danish cohort study has demonstrated that the increased use of thiopurines and biologics in IBD over time was associated with a persistent significant decrease in surgery rates, along with a significant decrease in the use of 5-ASA and corticosteroids [56]. The study, however, did not have the power to demonstrate a surgery-sparing effect of these newer medications [56]. The Swiss study by Safroneeva et al. [57] demonstrated that early treatment of CD patients with immunosuppressants and biologics was associated with reduced risk of developing bowel strictures, and early immunosuppressants reduced the risk of intestinal and perianal surgery. Therefore, further research with longer follow-up periods is needed.

### Strengths and limitations

The strength of the present study is the large number of patients included that enabled us to give a representative overview of the IBD situation in Switzerland. To the best of our knowledge, this is the first study to explore prevalence, mortality as well as health care costs and utilization for IBD patients treated in all settings in Switzerland. Moreover, the data weren’t collected by means of self-report and therefore results were not distorted due to recall bias.

One major limitation of this study is the fact, that we were lacking diagnosis information in the ambulatory setting. Since medication therapy is the main pillar in the treatment of IBD patients [20, 22, 58] the identification of patients via ATC codes seems natural. The combination of at least one claim for UC and CD and at least one pharmacy claim for 5-ASA, Immunosuppressants or biologics has been used in previous studies on the prevalence and health care costs of patients with CD and UC [5, 18]. Due to the selected ATC codes for the definition of IBD, patients with mild disease severity are likely to be underrepresented. This in turn may lead to an overestimation of the health care costs of IBD patients when compared with a non-IBD population as we more likely missed those cases incurring lower costs. Because of the known diagnostic delay distinctive of IBD, as well as the relapsing and remitting nature of IBD, the number of IBD patients may be even higher [59, 60]. Furthermore, this study is based on outpatient prescription drug dispending and does not consider medications applied during hospitalizations. Moreover, patients who consulted a pediatric gastroenterologist who is registered as a pediatrician rather than a gastroenterologist might be misclassified as not having IBD. This may lead to an underestimation of the prevalence of IBD, especially in younger patients.

A second major limitation is the fact that time trends are based on the analysis of three time points over a 5-year-period. One should therefore be careful drawing conclusions on time trends.

It is conceivable that further factors might influence the study findings, like the time of diagnosis or the treatment delay, which cannot be identified by means of the Helsana data [61]. In addition, we were not able to discriminate between health care costs and utilization that were solely attributable to IBD.

## Conclusions

Based on our analysis of three time points within 5 years, the prevalence rates of IBD seem to have increased in Switzerland. There was a shift from inpatient to outpatient costs in IBD patients, although no significant reduction in inpatient costs was observed between 2010 and 2014. Similarly, we found substantially higher medication costs in IBD patients treated with biologics that were not compensated by lower inpatient costs, compared with IBD patients without a prescription for biologics. However, more research with longer follow-up duration is needed to confirm these results.

Owing to new pharmacotherapies and changes in the management of the disease, there is a lack of research on the health care costs and utilization based on those pharmacotherapies used in clinical practice in patients with IBD. Further research is needed to demonstrate the burden of disease and the management of patients, separately for UC and CD, and to find the best possible pharmacologic intervention for each patient.

## Additional files

Additional file 1: Table S1. Age distribution of men and women in the Helsana cohort and the general Swiss population in 2010, 2012 and 2014. (DOCX 17 kb)

Additional file 2: Table S2. Median total health care costs (in Swiss Francs) of the IBD and the non-IBD sample extrapolated to the general Swiss population in 2010, 2012, and 2014, respectively. (DOCX 18 kb)

Additional file 3: Table S3. Health care costs (in Swiss Francs) of the IBD versus the non-IBD sample for the year 2014 (*n* = 1,125,050). (DOCX 16 kb)

Additional file 4: Table S4. Physician visits of the IBD versus the non-IBD sample for the year 2014 (*n* = 1,125,050). (DOCX 16 kb)

## Abbreviations

5-ASA: 5-aminosalicylic acid
ATC: Anatomical therapeutic chemical
CD: Crohn’s disease
CHF: Swiss Francs
CI: Confidence intervals
COPD: Chronic obstructive pulmonary disease
DRG: Diagnosis related group
HIV: Human immunodeficiency virus
IBD: Inflammatory bowel disease
ICD: International Classification of Diseases System
IQR: Interquartile range
OR: Odds ratio
PCG: Pharmacy-based cost group
SD: Standard deviation
TNF antagonist: Tumor necrosis factor alfa antagonist
UC: Ulcerative colitis
WHO: World Health Organization

## References

[1] Cleynen I, Boucher G, Jostins L, Schumm LP, Zeissig S, Ahmad T (2016). Inherited determinants of Crohn's disease and ulcerative colitis phenotypes: a genetic association study. Lancet.

[2] Conrad K, Roggenbuck D, Laass MW (2014). Diagnosis and classification of ulcerative colitis. Autoimmun Rev.

[3] Laass MW, Roggenbuck D, Conrad K (2014). Diagnosis and classification of Crohn's disease. Autoimmun Rev.

[4] Manninen P, Karvonen A-L, Huhtala H, Rasmussen M, Collin P (2010). The epidemiology of inflammatory bowel diseases in Finland. Scand J Gastroenterol.

[5] Kappelman MD, Rifas-Shiman SL, Kleinman K, Ollendorf D, Bousvaros A, Grand RJ, Finkelstein JA (2007). The prevalence and geographic distribution of Crohn's disease and ulcerative colitis in the United States. Clin Gastroenterol Hepatol.

[6] Bernstein CN, Wajda A, Svenson LW, MacKenzie A, Koehoorn M, Jackson M (2006). The epidemiology of inflammatory bowel disease in Canada: a population-based study. Am J Gastroenterol.

[7] Kappelman MD, Moore KR, Allen JK, Cook SF (2013). Recent trends in the prevalence of Crohn's disease and ulcerative colitis in a commercially insured US population. Dig Dis Sci.

[8] Lapidus A. Crohn's disease in Stockholm County during 1990-2001: an epidemiological update. World J Gastroenterol. 2006;12:75–81. 10.3748/wjg.v12.i1.75 PMC4077499 16440421

[9] Molodecky NA, Soon IS, Rabi DM, Ghali WA, Ferris M, Chernoff G (2012). Increasing incidence and prevalence of the inflammatory bowel diseases with time, based on systematic review. Gastroenterology.

[10] Zhai H, Liu A, Huang W, Liu X, Feng S, Wu J (2016). Increasing rate of inflammatory bowel disease: a 12-year retrospective study in NingXia, China. BMC Gastroenterol.

[11] Hwang C, Ross V, Mahadevan U (2012). Micronutrient deficiencies in inflammatory bowel disease: from a to zinc. Inflamm Bowel Dis.

[12] Fuller-Thomson E, Sulman J (2006). Depression and inflammatory bowel disease: findings from two nationally representative Canadian surveys. Inflamm Bowel Dis.

[13] Fuller-Thomson E, Lateef R, Sulman J (2015). Robust association between inflammatory bowel disease and generalized anxiety disorder: findings from a nationally representative Canadian study. Inflamm Bowel Dis.

[14] Walker JR, Ediger JP, Graff LA, Greenfeld JM, Clara I, Lix L (2008). The Manitoba IBD cohort study: a population-based study of the prevalence of lifetime and 12-month anxiety and mood disorders. Am J Gastroenterol.

[15] Burisch J, Jess T, Martinato M, Lakatos PL (2013). The burden of inflammatory bowel disease in Europe. J Crohns Colitis..

[16] Park KT, Bass D (2011). Inflammatory bowel disease-attributable costs and cost-effective strategies in the United States: a review. Inflamm Bowel Dis.

[17] van der Valk, Mirthe Emilie, Mangen M-JJ, Leenders M, Dijkstra G, van Bodegraven AA, Fidder HH, et al. Healthcare costs of inflammatory bowel disease have shifted from hospitalisation and surgery towards anti-TNFalpha therapy: results from the COIN study. Gut. 2014;63:72–9. 10.1136/gutjnl-2012-303376 23135759

[18] Kappelman MD, Rifas-Shiman SL, Porter CQ, Ollendorf DA, Sandler RS, Galanko JA, Finkelstein JA (2008). Direct health care costs of Crohn's disease and ulcerative colitis in US children and adults. Gastroenterology.

[19] Odes S, Vardi H, Friger M, Wolters F, Russel MG, Riis L (2006). Cost analysis and cost determinants in a European inflammatory bowel disease inception cohort with 10 years of follow-up evaluation. Gastroenterology.

[20] Dignass A, van Assche G, Lindsay JO, Lemann M, Soderholm J, Colombel JF (2010). The second European evidence-based consensus on the diagnosis and management of Crohn's disease: current management. J Crohns Colitis..

[21] Dignass A, Eliakim R, Magro F, Maaser C, Chowers Y, Geboes K (2012). Second European evidence-based consensus on the diagnosis and management of ulcerative colitis part 1: definitions and diagnosis. J Crohns Colitis..

[22] Dignass A, Lindsay JO, Sturm A, Windsor A, Colombel J-F, Allez M (2012). Second European evidence-based consensus on the diagnosis and management of ulcerative colitis part 2: current management. J Crohns Colitis..

[23] van Assche G, Vermeire S, Noman M, Amant C, Weyts E, Vleminckx A (2010). Infliximab administered with shortened infusion times in a specialized IBD infusion unit: a prospective cohort study. J Crohns Colitis..

[24] Schoepfer AM, Bortolotti M, Pittet V, Mottet C, Gonvers J-J, Reich O (2014). The gap between scientific evidence and clinical practice: 5-aminosalicylates are frequently used for the treatment of Crohn's disease. Aliment Pharmacol Ther.

[25] Braegger CP, Ballabeni P, Rogler D, Vavricka SR, Friedt M, Pittet V (2011). Epidemiology of inflammatory bowel disease: is there a shift towards onset at a younger age?. J Pediatr Gastroenterol Nutr.

[26] Juillerat P, Pittet V, Bulliard JL, Guessous I, Antonino AT, Mottet C (2008). Prevalence of inflammatory bowel disease in the Canton of Vaud (Switzerland): a population-based cohort study. J Crohns Colitis..

[27] Who Collaborating Center for Drug Statistics Methodology. Guidelines for ATC Classification and DDD Assignment 2015. 2014:1–288.

[28] Chini F, Pezzotti P, Orzella L, Borgia P, Guasticchi G (2011). Can we use the pharmacy data to estimate the prevalence of chronic conditions? A comparison of multiple data sources. BMC Public Health.

[29] Huber CA, Schneeweiss S, Signorell A, Reich O (2013). Improved prediction of medical expenditures and health care utilization using an updated chronic disease score and claims data. J Clin Epidemiol.

[30] Lamers LM, van Vliet RCJA (2004). The pharmacy-based cost group model: validating and adjusting the classification of medications for chronic conditions to the Dutch situation. Health Policy.

[31] Huber CA, Szucs TD, Rapold R, Reich O (2013). Identifying patients with chronic conditions using pharmacy data in Switzerland: an updated mapping approach to the classification of medications. BMC Public Health.

[32] Lumley T. Survey: Analysis of complex survey samples. 2014. http://www.R-project.org/.

[33] R Core Team. R: A Language and Environment for Statistical Computing. 2015. http://www.R-project.org/.

[34] Büsch K, Ludvigsson JF, Ekstrom-Smedby K, Ekbom A, Askling J, Neovius M (2014). Nationwide prevalence of inflammatory bowel disease in Sweden: a population-based register study. Aliment Pharmacol Ther.

[35] Hein R, Koster I, Bollschweiler E, Schubert I (2014). Prevalence of inflammatory bowel disease: estimates for 2010 and trends in Germany from a large insurance-based regional cohort. Scand J Gastroenterol.

[36] Pittet V, Juillerat P, Mottet C, Felley C, Ballabeni P, Burnand B, et al. Cohort profile: the Swiss inflammatory bowel disease cohort study (SIBDCS). Int J Epidemiol 2009;38:922–931. 10.1093/ije/dyn180 18782896

[37] Sulz MC, Siebert U, Arvandi M, Gothe RM, Wurm J, von Kanel R (2013). Predictors for hospitalization and outpatient visits in patients with inflammatory bowel disease: results from the Swiss inflammatory bowel disease cohort study. Eur J Gastroenterol Hepatol.

[38] Loftus EV, Clinical JR (2004). Epidemiology of inflammatory bowel disease: incidence, prevalence, and environmental influences. Gastroenterology.

[39] Bernstein CN, Blanchard JF, Rawsthorne P, Wajda A (1999). Epidemiology of Crohn's disease and ulcerative colitis in a central Canadian province: a population-based study. Am J Epidemiol.

[40] Manninen P, Karvonen AL, Huhtala H, Rasmussen M, Salo M, Mustaniemi L (2012). Mortality in ulcerative colitis and Crohn's disease. A population-based study in Finland. J Crohns Colitis.

[41] Romberg-Camps MJL, Bol Y, Dagnelie PC, Hesselink-van de Kruijs MAM, Kester ADM, Engels LGJB (2010). Fatigue and health-related quality of life in inflammatory bowel disease: results from a population-based study in the Netherlands: the IBD-South Limburg cohort. Inflamm Bowel Dis.

[42] Jess T, Loftus EV, JR, Harmsen WS, Zinsmeister AR, Tremaine WJ, Melton LJ3, et al. Survival and cause specific mortality in patients with inflammatory bowel disease: a long term outcome study in Olmsted County, Minnesota, 1940-2004. Gut 2006;55:1248–1254. 10.1136/gut.2005.079350 PMC1860022 16423890

[43] Jess T, Winther KV, Munkholm P, Langholz E, Binder V (2002). Mortality and causes of death in Crohn's disease: follow-up of a population-based cohort in Copenhagen County, Denmark. Gastroenterology.

[44] Hutfless SM, Weng X, Liu L, Allison J, Herrinton LJ (2007). Mortality by medication use among patients with inflammatory bowel disease, 1996-2003. Gastroenterology.

[45] Bewtra M, Kaiser LM, TenHave T, Lewis JD (2013). Crohn's disease and ulcerative colitis are associated with elevated standardized mortality ratios: a meta-analysis. Inflamm Bowel Dis.

[46] Card T, Hubbard R, Logan RF (2003). Mortality in inflammatory bowel disease: a population-based cohort study. Gastroenterology.

[47] Wei S-C, Lin M-H, Tung C-C, Weng M-T, Kuo J-S, Shieh M-J (2013). A nationwide population-based study of the inflammatory bowel diseases between 1998 and 2008 in Taiwan. BMC Gastroenterol.

[48] Sewell JL, Yee HF, JR. 13-year mortality trends among hospitalized patients with inflammatory bowel disease. BMC Gastroenterol 2012;12:79. 10.1186/1471-230X-12-79 PMC3441734 22734919

[49] AP Y, Cabanilla LA, EQ W, Mulani PM, Chao J (2008). The costs of Crohn's disease in the United States and other western countries: a systematic review. Curr Med Res Opin.

[50] Odes S. How expensive is inflammatory bowel disease? A critical analysis. World J Gastroenterol. 2008;14:6641–7. 10.3748/wjg.14.6641 PMC2773305 19034966

[51] Gibson TB, Ng E, Ozminkowski RJ, Wang S, Burton WN, Goetzel RZ, Maclean R (2008). The direct and indirect cost burden of Crohn's disease and ulcerative colitis. J Occup Environ Med.

[52] Hay JW, Hay AR (1992). Inflammatory bowel disease: costs-of-illness. J Clin Gastroenterol.

[53] Bickston SJ, Waters HC, Dabbous O, Tang BI, Rahman M (2008). Administrative claims analysis of all-cause annual costs of care and resource utilization by age category for ulcerative colitis patients. J Manag Care Pharm.

[54] Bassi A, Dodd S, Williamson P, Bodger K (2004). Cost of illness of inflammatory bowel disease in the UK: a single centre retrospective study. Gut.

[55] Nugent Z, Blanchard JF, Bernstein CNA (2010). Population-based study of health-care resource use among infliximab users. Am J Gastroenterol.

[56] Rungoe C, Langholz E, Andersson M, Basit S, Nielsen NM, Wohlfahrt J, Jess T (2014). Changes in medical treatment and surgery rates in inflammatory bowel disease: a nationwide cohort study 1979-2011. Gut.

[57] Safroneeva E, Vavricka SR, Fournier N, Pittet V, Peyrin-Biroulet L, Straumann A (2015). Impact of the early use of immunomodulators or TNF antagonists on bowel damage and surgery in Crohn's disease. Aliment Pharmacol Ther.

[58] Baumgart DC, Sandborn WJ (2012). Crohn's disease. Lancet.

[59] Vavricka SR, Spigaglia SM, Rogler G, Pittet V, Michetti P, Felley C (2012). Systematic evaluation of risk factors for diagnostic delay in inflammatory bowel disease. Inflamm Bowel Dis.

[60] Mozdiak E, O'Malley J, Arasaradnam R (2015). Inflammatory bowel disease. BMJ.

[61] Schoepfer AM, Dehlavi MA, Fournier N, Safroneeva E, Straumann A, Pittet V (2013). Diagnostic delay in Crohn's disease is associated with a complicated disease course and increased operation rate. Am J Gastroenterol.

