# Supplementary material for: Trends in prevalence, mortality, health care utilization and health care costs of Swiss IBD patients: a claims data based study of the years 2010, 2012 and 2014
Source: BMC Gastroenterol. 2017 Dec 2;17:138. doi: 10.1186/s12876-017-0681-y (PMC5712179; doi:10.1186/s12876-017-0681-y)
Supplement: Supplementary file 1 — Age distribution of men and women in the Helsana cohort and the general Swiss population in 2010, 2012 and 2014. (DOCX 17 kb) [file 12876_2017_681_MOESM1_ESM.docx]

| n (%) | **2010** | | **2012** | | **2014** | |
| --- | --- | --- | --- | --- | --- | --- |
|  | **Helsana** | **Switzerland** | **Helsana** | **Switzerland** | **Helsana** | **Switzerland** |
| **Total** | **1,170,913** | **7,870,100** | **1,163,351** | **8,039,100** | **1,151,373** | **8,237,700** |
| **Men** (total) | **564,357** | **3,877,400** | **562,960** | **3,968,500** | **557,253** | **4,073,900** |
| 1-17 years | 96,800 (17.2) | 746,300 (19.2) | 93,918 (16.7) | 750,400 (18.9) | 96,250 (17.3) | 761,200 (18.7) |
| 18-40 years | 170,268 (30.2) | 1,215,200 (31.3) | 170,602 (30.3) | 1,234,400 (31.1) | 164,188 (29.5) | 1,262,700 (31.0) |
| 41 - 60 years | 162,537 (28.8) | 1,164,400 (30.0) | 161,576 (28.7) | 1,196,200 (30.1) | 159,389 (28.6) | 1,226,000 (30.1) |
| > 60 years | 134,752 (23.9) | 751,600 (19.4) | 136,864 (24.3) | 787,500 (19.8) | 137,426 (24.7) | 824,000 (20.2) |
| **Women** (total) | **606,556** | **3,992,700** | **600,391** | **4,070,500** | **594,120** | **4,163,800** |
| 1-17 years | 91,555 (15.1) | 707,700 (17.7) | 88,553 (14.7) | 711,100 (17.5) | 90,741 (15.3) | 720,800 (17.3) |
| 18-40 years | 165,698 (27.3) | 1,193,800 (29.9) | 165,953 (27.6) | 1,207,400 (29.7) | 162,607 (27.4) | 1,232,900 (29.6) |
| 41 - 60 years | 164,963 (27.2) | 1,147,200 (28.7) | 161,003 (26.8) | 1,177,000 (28.9) | 158,334 (26.7) | 1,202,500 (28.9) |
| > 60 years | 184,340 (30.4) | 944,000 (23.6) | 184,882 (30.8) | 975,000 (23.9) | 182,438 (30.7) | 1,007,600 (24.2) |

Additional Table 1: Age distribution of men and women in the Helsana cohort and the general Swiss population in 2010, 2012 and 2014.
