# Supplementary material for: Trends in prevalence, mortality, health care utilization and health care costs of Swiss IBD patients: a claims data based study of the years 2010, 2012 and 2014
Source: BMC Gastroenterol. 2017 Dec 2;17:138. doi: 10.1186/s12876-017-0681-y (PMC5712179; doi:10.1186/s12876-017-0681-y)
Supplement: Supplementary file 2 — Median total health care costs (in Swiss Francs) of the IBD and the non-IBD sample extrapolated to the general Swiss population in 2010, 2012, and 2014, respectively. (DOCX 18 kb) [file 12876_2017_681_MOESM2_ESM.docx]

| Median (CI) | | **2010** | | | **2012** | | | **2014** | |
| --- | --- | --- | --- | --- | --- | --- | --- | --- | --- |
|  | **IBD** | | **Non-IBD** | **IBD** | | **Non-IBD** | **IBD** | | **Non-IBD** |
| **Total** | **5389** (5170-5607) | | **745** (742-749) | **6089** (5803-6374) | | **782** (778-786) | **6812** (6522-7102) | | **866** (862-870) |
| **Men,** total | **4856** (4489-5223) | | **454** (450-458) | **5454** (5100-5808) | | **481** (476-485) | **5970** (5603-6336) | | **544** (539-548) |
| 1-17 years | 5045 (2286-7805) | | 357 (352-362) | 6334 (3642-9027) | | 374 (369-380) | 6285 (1698-14,268) | | 405 (400-411) |
| 18-40 years | 3743 (3204-4282) | | 112 (109-115) | 3740 (3286-4195) | | 111 (108-113) | 4669 (3978-5360) | | 143 (140-147) |
| 41 - 60 years | 4189 (3773-4605) | | 494 (484-504) | 4907 (4389-5424) | | 505 (494-515) | 5238 (4716-5760) | | 563 (553-574) |
| > 60 years | 6584 (6082-7087) | | 2766 (2741-2792) | 7582 (68048360) | | 2840 (2813-2867) | 8262 (7469-9054) | | 3010 (2981-3038) |
| **Women,** total | **5679** (5371-5987) | | **1086** (1080-1093) | **6767** (6412-7121) | | **1134** (1127-1141) | **7599** (7115-8083) | | **1246** (1238-1253) |
| 1-17 years | 3699 (1304-6095) | | 348 (344-352) | 5101 (2021-8181) | | 364 (359-369) | 6587 (1629-11,545) | | 405 (340-410) |
| 18-40 years | 4498 (3917-5080) | | 744 (734-753) | 5642 (5056-6229) | | 768 (759-778) | 6466 (5584-7349) | | 873 (863-884) |
| 41 - 60 years | 5234 (4785-5683) | | 1210 (1196-1223) | 6058 (5473-6642) | | 1253 (1239-1267) | 7184 (6487-7881) | | 1362 (1347-1378) |
| > 60 years | 7081 (6547-7615) | | 3245 (3223-3268) | 8196 (7566-8827) | | 3350 (3323-3376) | 8649 (7902-9397) | | 3476 (3449-3503) |

Additional Table 2: Median total health care costs (in Swiss Francs) of the IBD and the non-IBD sample extrapolated to the general Swiss population in 2010, 2012, and 2014, respectively.
