# Supplementary material for: Trends in prevalence, mortality, health care utilization and health care costs of Swiss IBD patients: a claims data based study of the years 2010, 2012 and 2014
Source: BMC Gastroenterol. 2017 Dec 2;17:138. doi: 10.1186/s12876-017-0681-y (PMC5712179; doi:10.1186/s12876-017-0681-y)
Supplement: Supplementary file 3 — Health care costs (in Swiss Francs) of the IBD versus the non-IBD sample for the year 2014 (n = 1,125,050). (DOCX 16 kb) [file 12876_2017_681_MOESM3_ESM.docx]

Additional Table 3: Health care costs (in Swiss Francs) of the IBD versus the non-IBD sample for the year 2014 (n=1,125,050).

| Mean (SD, median) | IBD | non-IBD | *p*^a^ |
| --- | --- | --- | --- |
| Total | 12,734 (16,037, 6947) | 3793 (9370, 978) | <0.001 |
| Inpatient | 2253 (7973, 0) | 1015 (5153, 0) | <0.001 |
| Acute hospitals | 1873 (7268, 0) | 680 (4034, 0) | <0.001 |
| Psychiatric hospitals | 91 (1245, 0) | 91 (1663 ,0) | 0.02 |
| Others (e.g. nursing homes) | 290 (2120, 0) | 244 (2242, 0) | <0.001 |
| Outpatient | 5310 (6691, 3574) | 1936 (4101, 676) | <0.001 |
| Primary care physicians | 624 (776, 398) | 300 (507, 124) | <0.001 |
| Specialists | 1582 (1908, 1021) | 532 (1207, 40) | <0.001 |
| Others (e.g. paramedical) | 3104 (5794, 1531) | 1104 (3459, 194) | <0.001 |
| Medications | 5171 (8393, 1932) | 841 (3908, 131) | <0.001 |

^a^ *p-value* was assessed using Wilcoxon rank sum test.
