# Supplementary material for: Trends in prevalence, mortality, health care utilization and health care costs of Swiss IBD patients: a claims data based study of the years 2010, 2012 and 2014
Source: BMC Gastroenterol. 2017 Dec 2;17:138. doi: 10.1186/s12876-017-0681-y (PMC5712179; doi:10.1186/s12876-017-0681-y)
Supplement: Supplementary file 4 — Physician visits of the IBD versus the non-IBD sample for the year 2014 (n = 1,125,050). (DOCX 16 kb) [file 12876_2017_681_MOESM4_ESM.docx]

Additional Table 4: Physician visits of the IBD versus the non-IBD sample for the year 2014 (n=1,125,050).

| Median (IQR, mean) | IBD | non-IBD | *p*^a^ |
| --- | --- | --- | --- |
| Total number of visits^b^ | 16 (18, 21.3) | 6 (10, 10.5) | <0.001 |
| Primary care physicians | 5 (8, 7.5) | 3 (5, 4.4) | <0.001 |
| Specialists | 5 (9, 8.3) | 1 (4, 3.7) | <0.001 |
| Hospital outpatient visits | 2 (6, 5.5) | 0 (2, 2.4) | <0.001 |
| Number of different physicians contacted^b^ | 4 (3, 4.2) | 2 (2, 2.6) | <0.001 |

^a^ *p-value* was assessed using Wilcoxon rank sum test.

^b^ in patients with at least one physician visit.
